# Single-cell sequencing of PBMC characterizes the altered transcriptomic landscape of classical monocytes in BNT162b2-induced myocarditis

**DOI:** 10.3389/fimmu.2022.979188

**Published:** 2022-09-26

**Authors:** Nahee Hwang, Yune Huh, Seonghyeon Bu, Kyung Jin Seo, Se Hwan Kwon, Jae-woo Kim, Bo Kyung Yoon, Hyo-Suk Ahn, Sungsoon Fang

**Affiliations:** ^1^ Department of Biochemistry and Molecular Biology, Yonsei University College of Medicine, Seoul, South Korea; ^2^ Graduate School of Medical Science, Brain Korea 21 Project, Yonsei University College of Medicine, Seoul, South Korea; ^3^ Department of Medicine, Yonsei University College of Medicine, Seoul, South Korea; ^4^ Divison of Cardiology, Department of Internal medicine, The Catholic University of Korea, Uijeongbu St. Mary’s Hospital, Seoul, South Korea; ^5^ Catholic Research Institute for Intractable Cardiovascular Disease (CRID), College of Medicine, The Catholic University of Korea, Seoul, South Korea; ^6^ Department of Hospital Pathology, Uijeongbu St. Mary’s Hospital, College of Medicine, The Catholic University of Korea, Seoul, South Korea; ^7^ Department of Radiology, Kyung Hee University Medical Center, Seoul, South Korea; ^8^ Severance Biomedical Science Institute, Gangnam Severance Hospital, Yonsei University College of Medicine, Seoul, South Korea

**Keywords:** Coronavirus - COVID-19, single-cell RNA sequencing, monocyte - macrophage, vaccination, BNT162b2, myocarditis, transcriptome (RNA-seq)

## Abstract

The severe acute respiratory syndrome coronavirus 2 (SARS-CoV-2) has been the most dangerous threat to public health worldwide for the last few years, which led to the development of the novel mRNA vaccine (BNT162b2). However, BNT162b2 vaccination is known to be associated with myocarditis. Here, as an attempt to determine the pathogenesis of the disease and to develop biomarkers to determine whether subjects likely proceed to myocarditis after vaccination, we conducted a time series analysis of peripheral blood mononuclear cells of a patient with BNT162b2-induced myocarditis. Single-cell RNA sequence analysis identified monocytes as the cell clusters with the most dynamic changes. To identify distinct gene expression signatures, we compared monocytes of BNT162b2-induced myocarditis with monocytes under various conditions, including SARS-CoV-2 infection, BNT162b2 vaccination, and Kawasaki disease, a disease similar to myocarditis. Representative changes in the transcriptomic profile of classical monocytes include the upregulation of genes related to fatty acid metabolism and downregulation of transcription factor AP-1 activity. This study provides, for the first time, the importance of classical monocytes in the pathogenesis of myocarditis following BNT162b2 vaccination and presents the possibility that vaccination affects monocytes, further inducing their differentiation and infiltration into the heart.

## Introduction

The severe acute respiratory syndrome coronavirus 2 (SARS-CoV-2) pandemic has been a global threat for more than two years. However, as a result of scientific research, the world has succeeded in the development of messenger RNA (mRNA)-based vaccines based on novel technologies with unprecedented speed. Fortunately, Pfizer-BioNTech BNT162b2 injection showed high protection (>95%) against SARS-CoV-2 infection (1, 2) which led to its widespread use with more than 55 million shots given in the United States. However, recent studies have shown that BNT162b2 vaccination is associated with an increased risk of myocarditis, although the mechanism of action remains unknown (3, 4).

Peripheral immune activity is closely associated with the inflammatory response. For example, SARS-CoV-2 infection leads to reconfiguration of the peripheral immune cell phenotype (2). Characteristic immune cell phenotypes in patients hospitalized for the coronavirus disease 2019 (COVID-19) include HLA class II downregulation and type I interferon-driven inflammatory gene activation in monocytes (2). There have also been attempts to understand the changes in systems immunology after BNT162b2 vaccination by analyzing single-cell RNA sequencing data of the peripheral blood mononuclear cells (PBMCs), which showed a correlation of the monocyte-related signature with the neutralizing antibody level in response to the SARS-Cov-2 B.1.351 variant (5). Thus, it is noteworthy to investigate the changes in PBMCs, especially monocytes, in patients with inflammation in the cardiac muscle after COVID-19 vaccination.

An essential role of monocytes is to sense the environment and differentiate into macrophages in tissues, which proliferate upon inflammatory stimuli in the bone marrow (6) and their phenotypic and functional profiles change upon inflammatory signals and hence are important in disease progression (7). In cardiovascular diseases, an increased number of circulating monocytes is observed in patients with acute myocardial infarction (8, 9). Considering the importance of monocytes in acquired immunity after vaccination (10) including BNT162b2 vaccination (5), a thorough understanding of monocytes in BNT162b2-induced myocarditis (BNT162b2-MyoC) cases would give a new viewpoint regarding the pathogenesis of the event and provide biomarkers to rule out people at high risk in advance from vaccination.

In this study, we present a time series analysis of the transcriptomic changes in the peripheral immune landscape, focusing on monocytes, with single-cell RNA sequencing analysis of PBMCs from patients with BNT162b2-MyoC. Moreover, to evaluate the differences in transcriptomic profiles with those in similar conditions, we compared the gene signatures of monocytes in BNT162b2-MyoC with signatures under various conditions, including SARS-CoV-2 infection, BNT162b2 vaccination, and Kawasaki disease, a hyper-inflammatory disease similar to myocarditis (11). As a result, we identified distinct gene signatures of monocytes in patients with BNT162b2-MyoC, which include upregulation of the fatty acid metabolism pathway, downregulation of JUN/FOS activity, and dynamic changes in intercellular interactions with other immune cell types.

## Material and methods

### Ethics statement

The study was conducted in accordance with the Declaration of Helsinki and approved by the Institutional Review Board (IRB) of Uijeongbu St. Mary’s Hospital (UC19TIDE0142). Written informed consent was obtained from the participants.

### PBMC isolation

PBMC isolation was carried out on the day of blood collection from the patient. The blood in the EDTA tube was mixed with PBS in a 1:1 ratio. The mixture of blood and PBS was transferred to a Leucosept tube and centrifuged at a speed of 1000 g for 15 minutes at room temperature. Only the supernatant was moved to a 50ml conical tube. In order to get only the cells, centrifugation was performed at 400g for 10 minutes at room temperature. After aspirating supernatant, the cells washed twice.

After counting, the cells were resuspended in a stock solution (10% DMSO in fetal serum) and placed in a cell container in a deep freezer at -80°C for 24 hours. Finally, the stocks were stored in a liquid nitrogen tank.

### Chromium next GEM single Cell 5′v2 (dual index)

To get information on cell preparation, we used the LUNA-FL™ automated fluorescence cell counter (Logos Biosystems, Korea) to consult ‘the 10x Genomics Single Cell Preparation Guide’ and ‘the Cell Preparation Guide’ (documents CG00053 and CG000126, respectively).

Libraries were prepared using Chromium Single Cell 5’ Reagent Kits User Guide (v2 Chemistry Dual Index) (documents CG000331). In short, target cell count of 10,000 was achieved by diluting the cell suspension in nuclease-free water. After mixing with the master mix, the cell suspension was loaded with Single Cell 5′ Gel Beads and Partitioning Oil into a Next GEM Chip K. The single cells’ RNA transcripts were uniquely barcoded and reverse-transcribed within droplets. The cDNA products were pooled, and concentrated by the polymerase chain reaction (PCR) amplification.

In the case of the 5′ gene expression library, the cDNA pool undergoes an end repair process, the addition of a single ‘A’ base, and ligation of the adapters. The products were then purified and enriched by PCR to create a 5′ gene expression library. The purified libraries were quantified using qPCR according to the qPCR Quantification Protocol Guide (Kapa Biosystems, USA) and qualified using an Agilent Technologies 4200 TapeStation (Agilent Technologies, USA). The libraries were then sequenced using the HiSeq platform (Illumina, USA) according to the read length provided in the user guide.

### Single cell RNA-seq

The fastq files from single-cell 5′ profiling was further analyzed with the 10X Genomics CellRanger software (v.6.1.1). The 5′ transcriptome profiling was conducted with the Cellranger multi command. As the gene expression reference, the latest version of the human reference gene (GRCh38) was used. The expected cell number was 10,000. The R package Seurat (4.0.2) was used to create the object for further analysis (12).

Single cell RNA seq analysis of PBMCs were assayed exactly as previously described in the previous study (13).

### Quality control, data integration, and clustering

Along with our dataset, the dataset from our previous study, which is available in the Sequence Read Archive (SRA) under accession number (SRR18209602 and SRR18209603) and public datasets (GEO: GSE150728, GSE171964, and GSE167029) were used for bioinformatics analysis. The R package Seurat (4.0.2) was used for quality control, clustering, and differential gene expression analysis. The same quality control methods were used for all datasets used in this study Only cells with more than 100 features and less than 20% mitochondrial genes were used for the analysis. The filtered data were normalized using the NormalizeData function. “LogNormalize” method was used with a scale factor of 10,000. We then identified the variable genes on which the data were scaled. Variable genes were computed using the FindVairableFeature function. The “vst” selection method was used with 2,000 features. The data were scaled using the ScaleData function based on the computed variable genes. The RunPCA function was used based on the identified variable genes to identify the principal components (PCs). The first 50 PCs of the dataset were used for further clustering analysis. The RunUMAP function was used with the 50 most statistically significant PCs to infer the Uniform Manifold Approximation and Projection (UMAP) coordinates.

The FindNeighbors function was used for a shared nearest-neighbor graph (SNN) construction on the UMAP coordinates. SNN modularity optimization on the constructed SNN graph was performed using the FindClusters function to determine clusters.

### Datasets combination for meta-analysis

Individual Seurat objects were integrated with the filtered data using the R package Seurat. The SelectIntegrationFeatures and FindIntegrationAnchors functions were utilized to compute the integration anchors. Integration features are those that are consistently variable in all the datasets on which integration anchors can be established. Then, the IntegrateData function was used with the computed anchors to integrate the different Seurat datasets. After successfully merging the datasets, R Package Harmony (0.1.0) (14) was used on the integrated object for batch-effect correction. The RunHarmony function was used to integrate variances originating from different data sources and to create harmony embeddings. Clustering was conducted based on the updated harmony embeddings. Consequently, an integrated Seurat object with 35390 cells and 22 clusters was created.

We used these functions to integrate a single-cell-transcriptome, which analyzed PBMCs from patients with BNT162b2-induced myocarditis in the late recovery stage (92 days after vaccination), and the previously profiled transcriptome of PBMCs from the same patients in BNT162b2-induced myocarditis stage and early recovery stage (Day 16 and 21).

### Measuring differential gene expression and cluster annotation

Seurat FindAllMarkers with the default Wilcoxon rank sum test function was used to identify cluster markers. Genes whose logFC value was higher than 0.25 compared to that of the rest of the clusters were selected as cluster markers. The cell types for each cluster were manually annotated by comparing the selected cluster markers with reference genes. The reference genes used to determine the clusters are listed in **Supplemental Figure 1**. Visualizing Cell-to-Cell Communication Network

Intercellular communication was analyzed using the R package CellChat (1.1.3) (15). Interactions between cell clusters were computed based on the ligand−receptor pairs stored in the reference database CellChatDB. The communication probability of a specific signaling pathway level was computed based on the upregulated and downregulated ligand−receptor genes per cell group. The computeCommunProb function with default “trimean” truncated mean option was used for predicting communication probability of each signaling pathway. Significant computed signaling pathways were ranked with the rankNet function by measuring differences in overall information flow between the two conditions. The number and strength of interactions between the cell groups were visualized using the netVisual_aggregate function in circular plots.

### Gene pathway analysis

To measure differential gene expression, the Seurat FindMarkers function was used with the default Wilcoxon rank sum test method. The integrated Seurat object was split into multiple objects, each containing only one particular cell type. Differentially expressed genes (DEGs) between the two conditions from each Seurat object were used for further downstream analyses. Only genes with logFC > 0.15 and p < 0.01 were used.

Pathway analysis of the selected genes was performed using the R packages PROGENy (1.15.1) (16) and Enrichr (3.0.) (17) and it was conducted based on perturbation−response genes using the progeny function. Pathway activity scores for each condition were computed based on the perturbed genes. Phenotype-based permutation tests were conducted using the Enrichr function based on selected pathway databases. The databases used were as follows: 2021 Kyoto Encyclopedia of Genes and Genomes (KEGG) human biological pathways, 2021 Gene Ontology (GO) molecular function, and 2021 GO biological process. Only pathways with p < 0.05 were selected.

Next, the software Gene Set Enrichment Analysis (GSEA) (4.22) (18) was used to compute the normalized enrichment scores of the selected pathways. DE gene sets were converted into GCT file formats for software inputs. Biological pathways in which genes appeared at a higher frequency than expected were considered enriched. The results were visualized using default enrichment plots. Enrichment scores with p < 0.05 were considered significant.

### Measuring transcription factor activity

TF activity was inferred using the R package SCENIC (1.2.4) (19) based on the underlying gene regulatory network (GRN) in each cell. First, candidate target genes co-expressed with TFs were identified using GENIE3 as the standard SCENIC workflow in R. Target genes were further narrowed down by validating whether they were actually significantly enriched with the binding motifs of the candidate TFs. Putative binding motifs for each TF, stored in the RcisTarget database, were used for validation. Only regulatory modules with statistically significant motif enrichment were selected as significant regulons. The AUCell algorithm was then used to create a binarized expression matrix based on the activity scores of the inferred regulons. The algorithm produces an expression matrix with reduced dimensionality that presents the most active regulon activity score for each cell. The cells were further clustered based on the most active GRN. The clustered cell coordinates were then projected onto the existing UMAP.

The Curated human TF database, TRRUST (version 2) (20) was used to validate the downstream target genes of specific TFs. Briefly, TRRUST is a TF regulatory network database that stores 8,015 interactions between 748 TFs and their 1,975 target genes were based on manual curation implemented using a text-mining approach. Candidate regulatory target genes of activation protein-1 (AP-1) TF were identified using the TRRUST database. plots.

### Patient information

Additional patient information is accessible in **Supplementary Table 1** and from the paper that provided each dataset (2, 5, 11, 13).

## Results

### Time series analysis of single-cell RNA sequencing data of PBMCs of a patient with BNT162b2-MyoC

We previously profiled the single-cell transcriptome of PBMCs at two time points: the severe myocarditis stage (16 days after vaccination) and the early recovery stage (day 21) from a patient with myocarditis induced by BNT162b2 vaccination (SRR18209602 and SRR18209603). And more detailed clinical information of the patients is accessible in our previous study (13). To explore the transcriptomic characteristics of immune cells during BNT162b2-MyoC in more detail, we performed additional blood collection for single-cell RNA sequencing analysis from the same patient at the time point of late recovery phase (day 93) (**Supplementary Figure 1A**).

**Figure 1:**
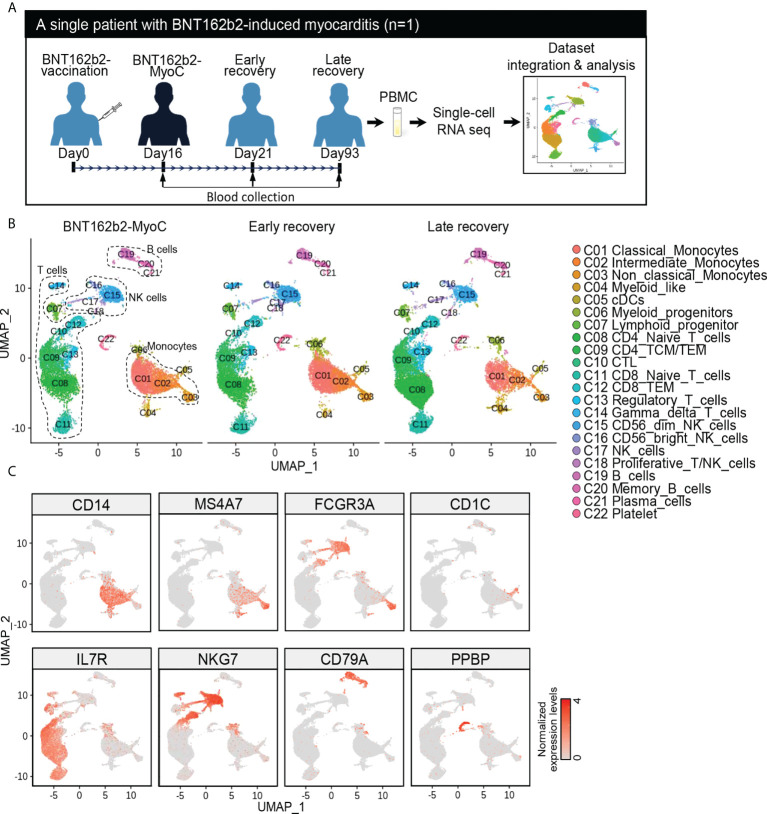
Time Series Analysis of Single-Cell RNA Sequencing Data of PBMCs of a Patient with BNT162b2-MyoC. **(A)** Overview of single-cell RNA-seq analysis of the PBMCs from patient in the stage of myocarditis(day16), early(day21), and late(day93) recovery stage after BNT162b2 administration, respectively. The samples were performed single-cell RNA-seq and integrated to one dataset with Harmony. **(B)** The dimensional reduction is performed with the uniform manifold approximation and projection (UMAP). Each dot represents a cell in each corresponding group, and is colored according to cell type The cells are pooled across all patients and separated by conditions: Myocarditis after BNT62b2-administration (BNT162b2-MyoC, left), early (middle), and late (left) recovery stages. Total number of cells per group: Myocarditis (n = 10,580cells), Early recovery (n = 11,745cells) and Late recovery(n = 13,065cells). **(C)** Normalized expression levels of marker genes for each immune cell type on UMAP plots. CD14 (Classical monocytes and Intermediated monocytes), MS4A7 and FCGR3A (Intermediated monocytes and Non-classical Monocytes), CD1C (Dendritic cells (cDCs)), IL7R (T cells), NKG7(Natural killer cells (NK cells)), CD79A (B cells), and PPBP (Platelets).

First, we integrated the newly analyzed data (day 93) with the previous dataset (SRR18209602 and SRR18209603) (13) (**Figure 1A**). After all datasets were completely harmonized, cells in the dataset were visualized in two-dimensional space using UMAP analysis (**Figure 1** and **Supplementary Figure 2A**). Single-cell sequencing analysis showed 22 clusters, and the cell types were initially identified with scCATCH and further analyzed using the expression patterns of marker genes for each immune cell type (**Figure 1C** and **Supplementary Figures 2B, C**), respectively. The cell cluster of classical monocytes was verified to have distinctively upregulated expression of CD14 (**Figure 1C**).

### Overall transcriptomic profiles of classical monocytes in BNT162b2-MyoC stage

**Figure 2:**
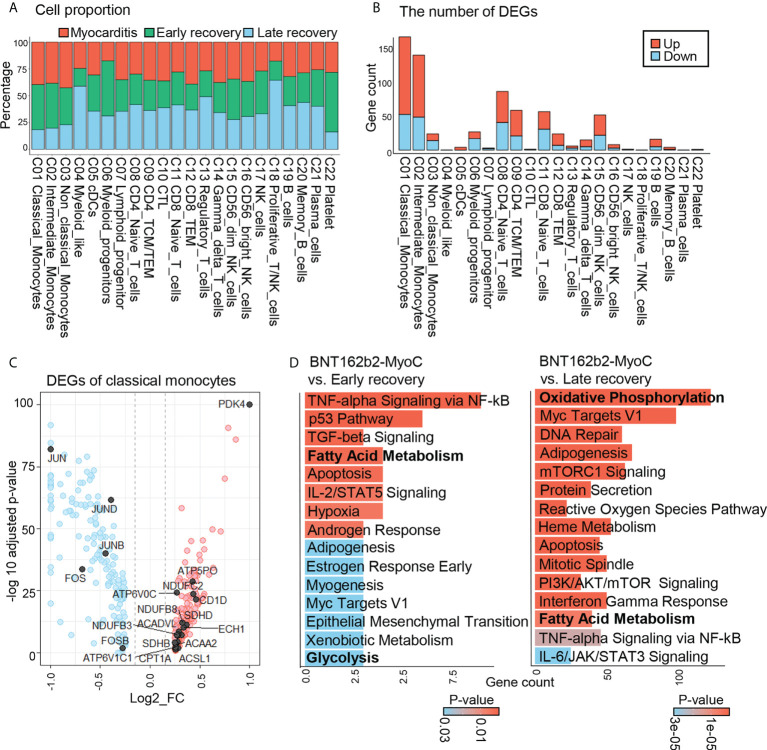
Overall Transcriptomic Profiles of Classical Monocytes in BNT162b2-MyoC Stage. **(A)** A Bar plot showing the proportion of each cell types derived from the patient in BTN162B2-MyoC, early recovery and late recovery phase, respectively. **(B)** Numbers of differentially expressed genes (DEGs) within each cell type in the patient in the stage of BTN162B2-MyoC compared to early recovery (p-value< 0.01, log2 fold-change (log2 FC) ≥ 0.15 or ≤ −0.15). Down: down-regulated; Up; up-regulated. **(C)** Gene expression ratio (log2 FC) of classical monocytes in the patients in BTN162B2-MyoC state versus in other groups (early and late recovery state) (horizontal axis) plotted against –Log adjusted p-value (vertical axis), showing DEGs: highly expressed (red) and lowly expressed in BNT162b2-MyoC (blue). (p-value < 0.01, log2 FC ≥ 0.15 or ≤ −0.15). **(D)** Gene set enrichment analysis with ‘Enrichr’ for up- regulated DEGs of classical monocytes in PBMCs from the patient in BNT162b2-MyoC state versus in early and late recovery stages, respectively. MSigDB_Hallmark_2020_terms are classified. P-value (color) and gene count (vertical axis) of the 20 most significant GO terms are shown.

After annotating each cluster, we calculated the proportion of cells originating at each time point for each cell cluster (**Figure 2A**). The presence of each cluster at every time point implies that the data are well-integrated and highly reproducible (21). However, there were differences in terms of cell proportions; the cell number across all clusters at the time of myocarditis was similar to that in the early recovery state but different from that in the late recovery state. Among all clusters, classical, intermediate, and non-classical monocytes showed the greatest increase in terms of cell number at the time of myocarditis compared to cell numbers in the late recovery state (**Figure 2A**). When PBMC at the time of myocarditis is compared with PBMC at early recovery state, there was minimal change in terms of cell number (**Figure 2A**); however, transcriptomic profiles were different judged by the number of differentially expressed genes (DEGs) (**Figure 2B** and **Supplementary Figure 3A**). As in the comparison of cell numbers, classical and intermediate monocytes showed the greatest number of DEGs.

Classical monocytes account for a major proportion of immune cells infiltrating the cardiac tissue in myocarditis (22). After infiltration, they differentiate into inflammatory macrophages and release pro-inflammatory cytokines, such as TNF-α and IL-6, which contribute to T cell activation and damage to the tissues. “Indeed, through endomyocardial biopsies of the patient of our dataset, we confirmed extensive myocardial tissue infiltration of macrophages during BNT162b2-induced myocarditis. **Supplementary Figures 1B, C**) (13).” In this study, we explored the transcriptomic features of classical monocytes over the course of time in patient with BNT162b2-MyoC. First, we figure out gene signatures of monocytes at the time of myocarditis. Compared to the other two time points, monocytes in myocarditis showed upregulation of genes related to fatty acid metabolism, such as *PDK4*, *ACSL1, ACSL4, CPT1A, ACADVL, ECHS1*, and *ACAA1*, and downregulation of genes related to the AP-1 complex (*JUN, JUNB, JUND, FOS*, and *FOSB*) (**Figure 2C**). Next, we conducted pathway analysis with Enrichr (dataset: MSigDB Hallmark 2020) for the pathway analysis. Consistently, the fatty acid metabolism-related pathway was upregulated at the time of myocarditis compared to the recovery states (**Figure 2D**). We also estimated the activity of signaling pathways in classical monocytes using PROGENy, which infers pathway activity based on the expression level of responsive genes (16). The results show that the P53, TGF-β, and MAPK pathways were activated in classical monocytes treated at the time of myocarditis. In contrast, the TNF-α, JAK-STAT, NF-κB, EGFR, and PI3K pathways were downregulated (**Supplementary Figure 3B**).

### JUN, FOS are significantly down-regulated in classical monocytes in BNT162b2-MyoC stage

**Figure 3:**
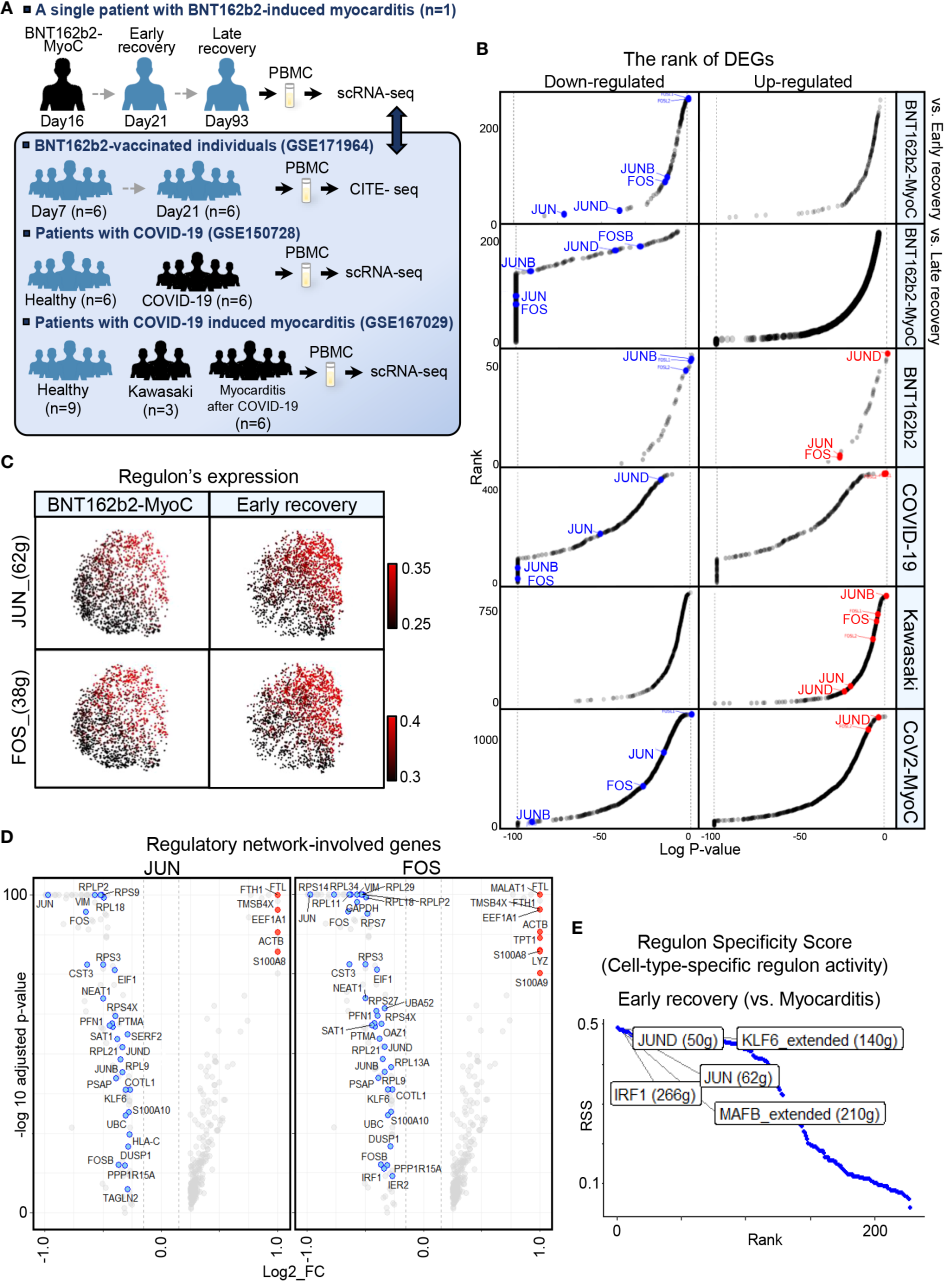
JUN, FOS are the Most Down-regulated in Classical Monocytes in BNT162b2-MyoC Stage. **(A)** A schematic of the experimental pipeline. PBMCs were obtained from the BNT162b2 vaccinated patient in the stage of myocarditis (day16), early recovery(day21), and late recovery(day93), respectively. The samples were performed single-cell RNA-seq and integrated to one dataset with Harmony. Publicly available datasets of PBMCs from BNT162b2-vaccinated individuals day7 and day21 after primary vaccination (GSE171964), PBMCs from healthy individuals and patients with COVID-19(GSE158055), and PBMCs from control group, patients with Kawasaki, and patients with COVID-19-induced myocarditis (CoV2-MyoC) were explored to understand the transcriptomic features of classical monocytes in more depth. **(B)** Log adjusted p-value (vertical axis) of DEGs of Classical monocytes in BNT162b2-MyoC plotted against the rank of average expression levels (vertical axis). The expression of AP-1(composed with JUN, JUNB, JUND, FOS, and FOSB. **(C)** UMAP showing expression levels of regulons of JUN and FOS in classical monocytes in BNT162b2-MyoC and in early recovery stage. **(D)** Volcano plots showing genes involved in regulatory network of JUN (left) and FOS (right) in M1 in DEGs of BTN162B2-MyoC group (horizontal axis) **(E)** Regulons with top 5 cell-type activity in classical monocytes in early recovery stage compared to BNT162b2-MyoC.

To elucidate the characteristics of classical monocytes in patients with BNT162b2-MyoC in more detail, we performed comparative analysis with various datasets: single-cell RNA sequencing data of PBMCs from seven patients with COVID-19 and six healthy individuals (GSE150728) (2), CITE-sequencing data of PBMCs from six vaccinated individuals without side effects (GSE171964) (5), and single-cell RNA sequencing data of PBMCs from young patients with Kawasaki disease or COVID-19-induced myocarditis (CoV2-MyoC) (GSE167029) (11) (**Figure 3A** and **Supplementary Figures 4A−C**). The GSE167029 dataset contains single-cell transcriptomic profile of nine healthy individuals as a control group, six patients with CoV2-MyoC, and two patients with Kawasaki disease. Details of each sample can be found in the paper that provides each dataset. Also, to reduce the gap between time points of the blood collection from patients with BNT162b2 induced myocarditis (16, 21, and 93 days after 1st vaccination, respectively) and BNT162b2- vaccinated-individuals (GSE171964) as much as possible, we selected group of 7 days and 21days after the 1st vaccination (among 0,1,2,7 and 21 days after 1st and 2nd vaccination).

AP-1 is a TF complex consisting of JUN, JUNB, JUND, FOS, and FOSB. The AP-1 complex is known to affect various cellular processes, such as proliferation, differentiation, apoptosis, and immune cell activation, and macrophage differentiation (23–25). Therefore, AP-1 is thought to be associated with the immune activity of classical monocytes in patients with inflammation. In addition, mRNA levels of AP-1 especially FOS and FOSB, were high in monocytes from our dataset (**Supplementary Figure 5A**). The expression level of AP-1 in classical monocytes was lower in BNT162b2-MyoC state than that in the early and late recovery states (**Figures 2C**, **3B** and **Supplementary Figure 5B**). Although all of the patients were with myocarditis, the classical monocytes in patients in each dataset showed different AP-1 activities.

For other disease conditions, AP-1 expression increased in vaccinated individuals and patients with Kawasaki disease but decreased in patients with COVID-19 and CoV2-MyoC (**Figure 3B** and **Supplementary Figure 5B**). To examine the regulatory activity of AP-1 in classical monocytes in our dataset, we analyzed gene regulatory network *via* SCENIC function. As expected, AP-1 regulon activity was downregulated in classical monocytes in BNT162b2-MyoC state and increased gradually at the patient recovered (**Figure 3C** and **Supplementary Figure 5C**). In addition, genes involved in the regulatory network of JUN and FOS were mostly downregulated during myocarditis in classical monocytes (**Figure 3D**). Ultimately JUN/JUND was one of the regulons that showed the greatest up-regulation at the time of early recovery (**Figure 3E**).

### Fatty acid metabolism is highly up-regulated in classical monocytes in BNT162b2-MyoC stage

**Figure 4:**
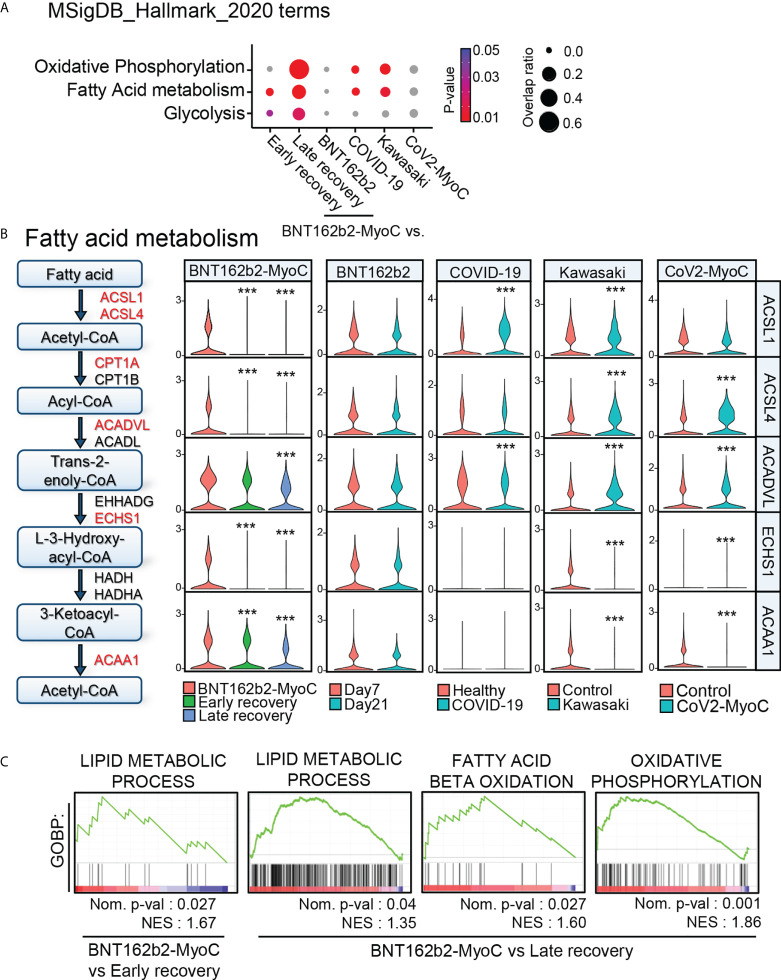
Fatty Acid Metabolism is Highly Up-regulated in Classical Monocytes in BNT162b2-MyoC Stage. **(A)** Enrichment analysis with cellular metabolism-related Hallmark gene sets for DEGs of classical monocytes in each dataset. **(B)** A diagram of fatty-acid metabolism-pathway and violin plots showing the expression levels of the pathway-related genes in classical monocytes. *** p < 0.001; two-tailed t test. **(C)** GSEA analysis for the dataset of DEGs of classical monocytes in BNT162b2-MyoC group was conduct against GOBP_LIPID_METABOLIC_PROCESS, GOBP_OXIDATIVE_PHOSPHORYLATION, and GOBP_FATTY_ACID_BETA_OXIDATION gene sets, respectively. A positive enrichment score on the y axis indicates positive correlation with BNT162b2-MyoC group.

According to other studies, the metabolism of classical monocytes is closely related to their activation state during inflammation (26–28). By performing pathway analysis *via* Enrichr with MSigDB Hallmark 2020 gene sets (29), we revealed that the activity of pathways related to oxidative phosphorylation, fatty acid metabolism, and glycolysis was significantly increased during BNT162b2-MyoC (**Figures 2D** and **4A**). Thus, in contrast to JUN and FOS down-regulated at the time of myocarditis, metabolism-related genes were significantly up-regulated. In addition, classical monocytes of patients with COVID-19 and Kawasaki disease showed enrichment of fatty acid metabolism and glycolysis pathways compared to those of healthy individuals (**Figure 4A**) Since the metabolic profile of classical monocytes varied depending on the diseases, we explored fatty acid metabolism pathway in more depth. Next, we evaluated the expression of marker genes involved in fatty acid metabolism in classical monocytes. The expression of these genes gradually decreased over time. The pattern of changes in the expression levels of fatty acid metabolism-related genes was not identical in the analyzed disease conditions (**Figure 4B** and **Supplementary Figure 6A**). The expression of glycolysis-related genes was significantly reduced only in the late recovery group in our data, and the differences between BNT162b2-MyoC and the early recovery state were statistically insignificant. In other conditions, the expression levels of glycolysis-related genes were markedly increased in the monocytes of Kawasaki and patients with CoV2-MyoC (**Supplementary Figures 6A, B**). To clarify the transcriptomic characteristics of classical monocytes in the acute BNT162b2-MyoC state, we focused on the differences between BNT162b2-MyoC and the early recovery state rather than BNT162b2-MyoC and the late recovery stage. Therefore, we investigated fatty acid metabolism as a major metabolic characteristic of classical monocytes in acute BNT162b2-MyoC infection. We confirmed through Gene Set Enrichment Analysis (GSEA) that gene sets related to fatty acid metabolism were more enriched, with statistical significance, in BNT162b2-MyoC state (**Figure 4C**).

### CEBPB is the major transcription factor to mediate fatty acid metabolism in classical monocytes in BNT162b2-MyoC stage

**Figure 5:**
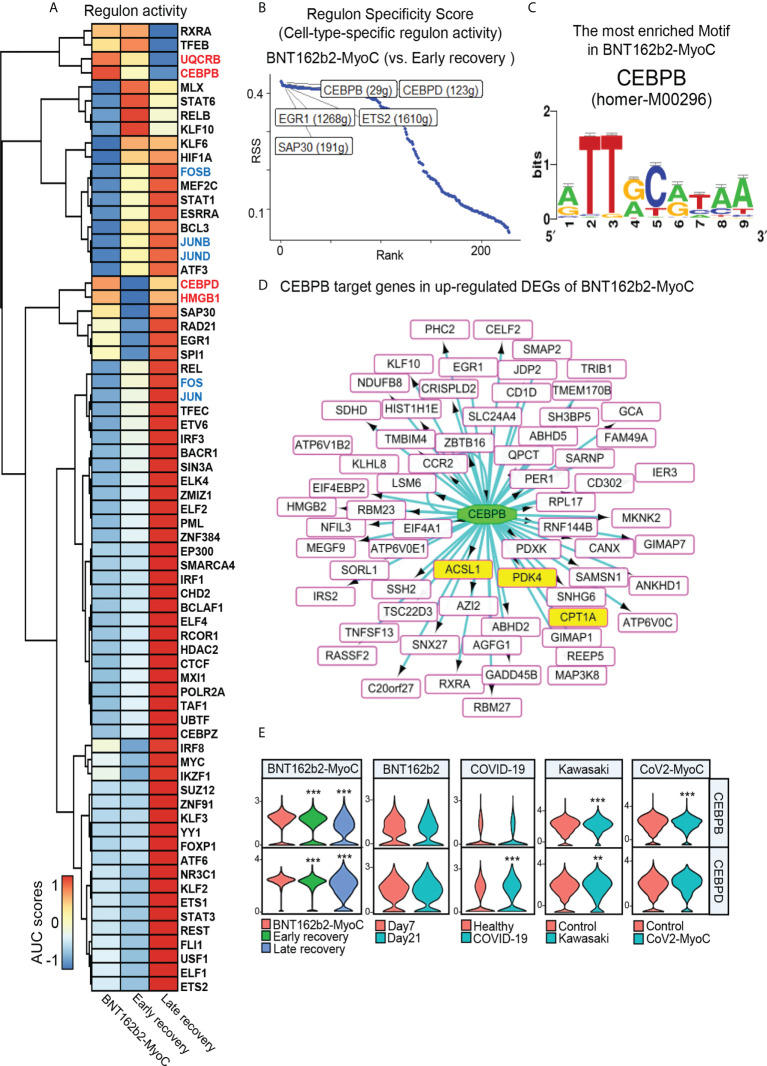
CEBPB is the Major Transcription Factor to Mediate Fatty Acid Metabolism in Classical Monocytes in BNT162b2-MyoC Stage. **(A)** A clustered heat map showing the area under the curve (AUC) scores of expression regulation by transcription factors (TFs), as estimated using SCENIC. **(B)** Regulons with top 5 cell-type-specific activity in classical monocytes in early recovery stage compared to BNT162b2-MyoC stage. **(C)** The CEBPB-bound motif, which is the most enriched in classical monocytes in BNT162b2-MyoC (NES score of 5.716 and 23 direct targets genes) predicted by iRegulon. **(D)** A Network plot showing target genes of CEBPB in up-regulated DEGs of classical monocytes in BNT162b2-MyoC. all target genes as squares. squares fille with yellow color are fatty acid metabolism related genes. CEBPB is drawn as a green ellipse. The interactions are shown with directed edges from the CEBPB to the target genes. **(E)** Normalized expression levels of CEBPB and CEBPD in classical monocytes in each group of datasets. ** p < 0.01; *** p < 0.001; two-tailed t test.

To predict the transcription factors associated with the changes in fatty acid metabolism-related genes, we explored the changes in regulon activities in classical monocytes at BNT162b2-MyoC stage. As a result of analysis using SCENIC, the regulons with the increase in the activity at BNT162b2-MyoC stage are as follows: CCAAT Enhancer Binding Protein Beta (CEBPB), Ubiquinol-Cytochrome C Reductase Binding Protein (UQCRB), CCAAT Enhancer Binding Protein Delta (CEBPD), and Sin3A Associated Protein 30 (SAP30) (**Figure 5A**). To explore regulon activity of the acute myocarditis phase, the regulon activity of BNT162b2-MyoC stage was compared only with early recovery stage. The regulon activity of CEBPB and CEBPD was the highest. (**Figure 5B**).

Also, we conducted iRegulon which is a gene-based tool to compute motif activity, predicting regulons, target genes and motifs from a set of co-expressed genes and maps gene-regulatory-network directly based on motif enrichment. As a result of an analysis with iRegulon with up-regulated DEGs of classical monocytes, it was confirmed that 11 CEBPB-binding motifs were enriched in classical monocytes at BNT162b2-MyoC stage. Especially, we found that homer-M00296, to which CEBPB binds, was the most enriched motif among 93 motifs enriched in classical monocytes at BNT162b2-MyoC stage (**Figure 5C** and **Supplementary Table 1**). For the patients with COVID-19, 7 CEBPB-binding motifs were enriched in classical monocytes. For the other datasets, the activity of CEBPB regulon was not high enough to be detected *via* iRegulon (**Supplementary Table 1**). CEBPB is a well-known transcription factor for mediating fatty acid metabolism (30–32). For example, in the case of nasopharyngeal carcinoma cells, CEBPB binds to PPAR coactivator-1α and promotes the transcription of CPT1A, which ultimately increased the level of fatty acid oxidation (30). Also, CEBPB controlled transcriptional regulatory networks important for inflammation and lipid metabolism in macrophages in mice during diet-induced inflammation (33). Interestingly, the predicted target genes of CEBPB by iRegulon included *CPT1A*, *ACSL1*, *PDK4* which were up-regulated at BNT162b2-MyoC stage in terms of expression level (**Figure 5D**). Finally, we investigated the transcriptional levels of CEBPB and CEBPD showing the highest level of regulon activity in BNT162b2-MyoC stage. For BNT162b2-MyoC dataset, the expression levels of both *CEBPB* and *CEBPD* gradually decreased with recovery. In the other datasets, *CEBPB* increased in the patients with Kawasaki and CoV2-MyoC, and *CEBPD* increased in the patients with COVID-19 and Kawasaki (**Figure 5E**).

Given that recent studies have highlighted the importance of fatty acid oxidation in immune activity of monocytes (34), especially during differentiation into macrophages (35–37), our study is meaningful in that it addressed the increased fatty acid metabolism in classical monocytes during BNT162b2-MyoC stage. Also, we suggest the possibility that CEBPB may play a critical role in this metabolic shift.

### Classical monocytes-mediated IL-16 and CXCL signals were enhanced during BNT162B2-MyoC stage

**Figure 6:**
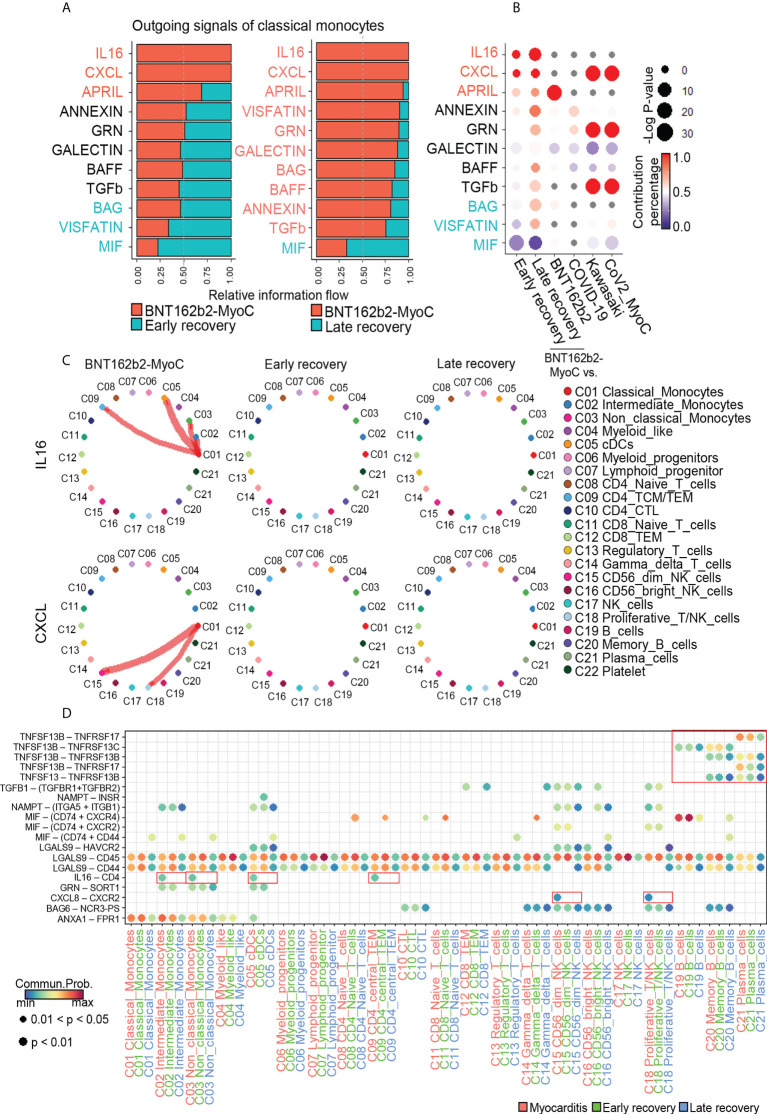
Classical monocytes-mediated IL-16 and CXCL signals were enhanced during BNT162B2-MyoC Stage. **(A)** Bar graphs showing the ranking of major outgoing signals of classical monocytes in the patients in BNT162B2-MyoC compared to early(left) and late(right) recovery stages, respectively. The rank of signals was based on differences in overall information flow of each group. **(B)** A dot plot showing signaling enrichment of classical monocytes in other datasets for the classical monocytes-specific outgoing signals specific in BNT162b2-MyoC stage. Grey dots indicate insignificant interactions. **(C)** Circle plots showing cell-to-cell network for increased outgoing signals of classical monocyte in BNT162b2-MyoC stage. Arrows and edge color indicate direction (source: target). Edge thickness indicates the sum of weight key signals between populations. **(D)** A dot plot showing the relative significance of each cell type for each signaling pathway based on the average expression of the ligand-receptor pair.

Metabolic changes in immune cells play an important role in immune responses *via* regulating inter/intra-cellular communication, such as cytokine secretion (38, 39). In the case of macrophages, it has been revealed that fatty acid oxidation activated their cytokine secretion, such as IL1b, IL4, IL18 and chemokine (C-X-C motif) ligand-1 (40). In this study, we examined the interaction of classical monocytes, which increased fatty acid metabolism in BNT162b2-MyoC stage, with analysis on the expression patterns of cytokine gene. CellChat was performed to analyze cell-to-cell communication. First, based on the differences in overall information flow within the network, we ranked the relative strength of the outgoing signals of classical monocytes at the time of myocarditis compared with signals in the early and late recovery states, respectively. In the BNT162b2-MyoC stage, IL-16, CXCL, and APRIL signals from classical monocytes increased compared to those in the early and late recovery stages. In contrast, a decreased level of TNF signal was shown at BNT162b2-MyoC stage (**Figure 6A**). Compared with other datasets, it was confirmed that IL-16 increased uniquely in the patient with BNT162b2-MyoC, and CXCL also increased in both patients with Kawasaki disease and CoV2-MyoC. APRIL signal increased the most in vaccinated individuals. On the other hand, the MIF signal, which decreased in our data, also decreased in patients with Kawasaki disease and CoV2-MyoC (**Figures 6B** and **Supplementary Figure 7A**). We confirmed that IL-16 signals flowed into intermediate monocytes, non-classical monocytes, dendritic cells (cDCs), and CD4^+^ TCM/TEM cells, while CXCL signals flowed into CD56^dim^ natural killer cells (CD56^dim^ NK cells) and proliferative T/NK cells. Both signals from classical monocytes were observed only in the BNT162b2-MyoC stage (**Figure 6C**). The APRIL signal is supplied to memory B and plasma cells (**Supplementary Figure 7B**). We explored the ligand–receptor pairs of outgoing signals of classical monocytes that increased in BNT162b2-MyoC. For the IL-16 signal, the ligand gene was *IL16* and the receptor gene was *CD4*. For the CXCL signal, the ligand gene was *CXCL8* and the receptor gene was *CXCR2*. For the APRIL signal, the ligand genes were *TNFSF13* and *TNFSF13B* and the receptor genes were *TNFRSF13B*, *TNFRSF13C*, and *TNFRSF17* (**Figure 6D** and **Supplementary Figure 7C**). It is worth noting the signal increase of IL-16 by classical monocytes, since this molecule is well-known for its chemoattractant activity on CD4^+^ immune cells. In addition, IL-16 induces the production of pro-inflammatory cytokines, such as IL-6, IL-15, and TNF-α (41, 42).

### Classical Monocytes receives Cytotoxic T cell-mediated incoming signals in BNT162b2-myocarditis stage

**Figure 7:**
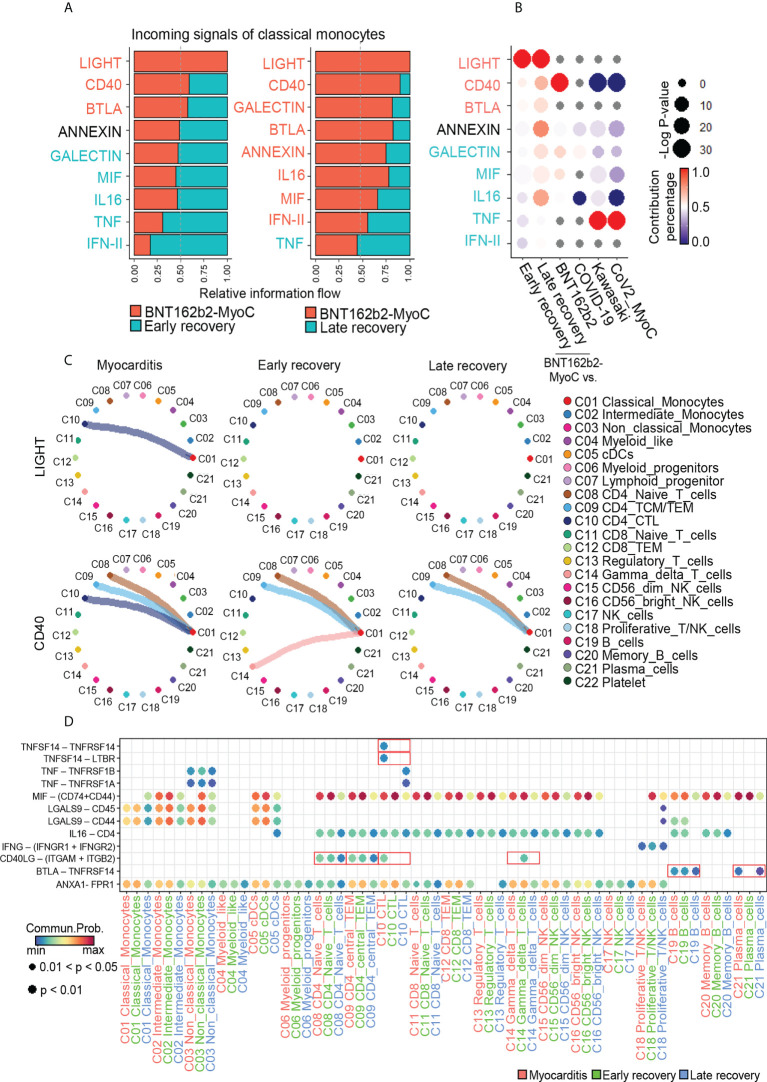
Classical Monocytes receives Cytotoxic T cell-mediated Incoming Signals in BNT162b2-myocarditis Stage. **(A)** Bar graphs showing the ranking of major incoming signals of classical monocytes in the patients in BNT162B2-MyoC stage compared to early(left) and late(right) recovery stages, respectively. The rank of signals was based on differences in overall information flow, which is calculated by the total weights in the cellular network, of each group. **(B)** A dot plot showing signaling enrichment of classical monocytes in other datasets for the classical monocyte-specific incoming signals in BNT162B2-MyoC stage. Grey dots indicate insignificant interactions. **(C)** Circle plots showing the intercellular network of increased incoming signals of classical monocytes in BNT162B2-MyoC stage. Arrows and edge color indicate direction (source: target). Edge thickness indicates the sum of weight key signals between populations. **(D)** A dot plot showing the relative significance of each cell type for each signaling pathway based on the average expression of the ligand-receptor pair.

Next, we studied signals incoming signal from classical monocytes. In the BNT162b2-MyoC state, the most enriched signals included LIGHT, CD40, and BTLA. Conversely, the signal enriched in both the early and late recovery states was TNF. (**Figure 7A**). Compared to other data, LIGHT and BTLA signals were enriched only in classical monocytes at the BNT162b2-MyoC stage. In the case of CD40, signaling enrichment was increased in vaccinated individuals but significantly decreased in patients with Kawasaki disease and CoV2-MyoC (**Figure 7B** and **Supplementary Figure 8A**). Next, we examined the contribution of each cell type to the incoming signals of classical monocytes in BNT162b2-MyoC (**Figure 7C**). For the LIGHT signal, cytotoxic T cells (CTLs) contributed the most in the increase in signaling enrichment in BNT162b2-MyoC. For the CD40 signal, CTLs, CD4^+^ central/effector memory T cells (CD4^+^ TCM/TEM), CD4^+^ naïve T cells, and gamma delta T cells contributed to classical monocyte signaling. Moreover, signals from CTL were only observed in the BNT162b2-MyoC group, and signals from both CD4^+^ TCM/TEM and CD4^+^ naïve T cells gradually decreased with the stage of BNT162b2-MyoC. Although the signals from gamma delta T cells in the treated stage increased, the overall CD40 inflow signal of classical monocytes was the strongest in the BNT162b2-MyoC stage (**Figures 7A, B**). In terms of BTLA signals, B cells and plasma cells were the dominant contributors to this interaction (**Supplementary Figure 8B**). Lastly, we confirmed that the ligand–receptor pairs correlated with the major incoming signals of classical monocytes in BNT162b2-MyoC state. For the LIGHT signal, the ligand was TNFSF14 and the receptors were TNFRSF14 and LTBR. For the CD40 signal, the ligand was CD40LG and the receptors were ITGAM and ITGB2. For the BTLA signal, the ligand was BTLA and the receptor was TNFRSF14. (**Figure 7D** and **Supplementary Figure 8C**).

Interestingly, TNFRSF14, a receptor of LIGHT signaling, is known to be involved in monocyte activity by inducing the secretion of pro-inflammatory cytokines, such as IL-8 and TNF-α. In addition, the interaction of CD40 ligand (CD40L; CD154) with Mac-1 (αMβ2, CD11b/CD18) on monocytes is known to induce adhesion and migration of classical monocytes and is related to pro-inflammatory functions (43). In summary, we inferred that interaction with CTL *via* LIGHT and CD40 signals is critical for the activity of classical monocytes during BNT162b2-MyoC.

Overall, our results showed significant changes in the expression of fatty acid metabolism-related genes and AP-1 activity over time in BNT162b2-MyoC patient. It is important to note that the expression patterns of metabolism-related genes vary depending on the cause of myocarditis. In addition, we established a framework for determining which types of cell-to-cell signals are critical for the activity of circulating monocytes in patients with BNT162b2-MyoC. Therefore, our study provides critical clues regarding the transcriptional profile of classical monocytes at the time of BNT162b2-MyoC.

## Discussion

Although vaccination is one of the most promising tools to fight COVID-19, it is associated with the risk of myocarditis. Among multiple side effects of BNT162b2 vaccination, myocarditis in particular has been extensively investigated, yet its exact pathogenesis still remains unknown (44). Although several case studies have reported clinical changes in BNT162b2-MyoC patients, our understanding of BNT162b2-MyoC is still limited mainly due to scarcity of tissue sampling and lack of empirical evidence (45). Moreover, based on published literature reviews (46, 47), among few empirical studies that were conducted with tissue samples from patients, there hasn’t been research that featured detailed transcriptomic profile of the harvested tissues. Myocarditis is a local inflammation of the myocardium. However, systemic immune modulation, such as cancer immunotherapy, has been reported to significantly increase myocarditis (48). Also, in the case of ST elevation myocardial infarction (STEMI), which is known to be major contributors of heart failures, transcriptional features of monocytes in PBMCs are in the spotlight as a biomarker for early prediction of heart failure (49). Therefore, understanding roles of monocyte is important in developing a systemic approach to understand the pathogenesis of myocarditis following BNT162b2 vaccination.

Therefore, it is important to develop a systemic approach to understand the pathogenesis of myocarditis following BNT162b2 vaccination. Here, we tracked transcriptomic profiles of the peripheral immune landscape of a patient recovered from a rare case of BNT162b2-MyoC and revealed that monocytes, which can be differentiated into macrophages in tissues, are the cell type showing one of the most dynamic changes.

Several studies on the metabolism of macrophages have proven the importance of metabolic pathways in monocyte-to-macrophage differentiation, macrophage activation, and polarization. Polarization of inflammatory macrophages increases the level of cellular glycolysis and fatty acid biosynthesis. However, polarization to anti-inflammatory macrophages upregulates the rates of oxidative phosphorylation and fatty acid oxidation (40). In addition, genes related to fatty acid and lipid metabolic processes, along with monocarboxylic acid and cellular ketone metabolic processes, are upregulated during monocyte-to-macrophage differentiation, highlighting the interconnection between immune cell function and metabolism (50–52). Here, investigation of the peripheral immune landscape of the patient in chronological order revealed that most differentially expressed genes are metabolic enzymes, including *PDK4*, the most upregulated gene in myocarditis, and genes involved in fatty acid metabolism. PDK4 is a mitochondrial enzyme that controls glycolytic flux into mitochondria. In addition, as the site of fatty acid oxidation, mitochondria govern the activation state of macrophages (53, 54) by modulating the metabolic profile of the cells. Thus, our study highlights the possibility of transcriptomic alteration in mitochondrial metabolism-related genes as a biomarker of the vaccination-induced myocarditis, which is diagnosed with the findings that show the infiltration of CD68+ cells in cardiac tissues.

In this study, we figured out CEBPB as a major transcription factor and the key regulator of fatty acid metabolism in classical monocytes during BNT162b2-myocarditis. Classical monocytes featured increased CEBPB activity in terms of both regulon activity and mRNA expression level. Also, we confirmed not only *PDK4*, but also *CPT1A* and *ACSL1*, which are known to be key regulators of fatty acid metabolism, are the target transcripts of CEBPB in classical monocytes in BNT162b2-myocarditis. In addition, the characteristics of classical monocytes during BNT162b2-myocarditis were further standardized by comparing with those of monocytes in similar conditions. Classical monocytes in the patient with acute stage of BNT162b2-myocarditis were similar to those in the patients with COVID-19 in terms of decreased level of JUN/FOS expression and increased regulon activity of CEBPB. Also, we confirmed that classical monocytes tended to rely more on glycolysis during BNT162b2-myocarditis compared to late recovery stage, in Kawasaki disease, and in COVID-19-induced myocarditis. Glycolysis as well as fatty acid metabolism is known to pro-inflammatory activation and differentiation of classical monocytes in diverse diseases (27, 55, 56). We tested if the changes in expression of metabolism-related genes are driven by extracellular stimuli such as cytokines from other immune cells. For example, transforming growth factor-β (TGF-β) signal is known to induce glycolysis *via* regulating the expression of the related genes in various cells; TGF-β induces expression of glucose transporter type 1 in Swiss 3T3 cells and glomerular mesangial cells, hexokinase 2 in articular chondrocytes and lung fibroblasts, and phosphofructokinase 2 in diverse cancer cells (57). In our study, the upregulation of glycolysis-related genes was confirmed in monocytes along with increased TGF-β-incoming signal.

Moreover, the metabolic enzymes involved in monocyte-to-macrophage differentiation are known to be closely related to several signaling pathways. For instance, human monocyte-derived macrophages are reported to upregulate metabolic-inflammatory transcriptional programs, including lipid metabolism and glycolytic pathways, upon activation by monosodium urate crystals, which induce inflammation without prior priming (58). The activation of these metabolic gene programs is attributed to the increased binding of JUN to the target promoters (58). Considering that monocytes in acute myocarditis show altered JUN/FOS activity, along with changes in the metabolism-related gene pathway, further investigation is required to determine whether monocytes in acute myocarditis resemble monocyte-derived macrophages activated without prior priming.

Also, other studies emphasized the importance of mobilization of proinflammatory monocytes to cardiac tissue for inflammatory cardiomyopathy disease. For example, CCR2–CCL2 and CX3CR1-CX3CL1 axis were associated with the recruitment of monocyte to cardiac tissue during myocarditis. Therefore, intercellular interactions of monocytes before penetrating cardiac tissue can be important biomarkers of myocarditis (22). In intercellular communication in this study, the increase in IL-16 signal outflow and LIGHT signal inflow were unique features of classical monocytes in BNT162b2-myocarditis stage. On the other hand, the increase in APRIL signal outflow and CD40 signal inflow from classical monocytes was similar to that of vaccinated individuals. And the increase in CXCL signal outflow was similar to that of patients with Kawasaki and COVID-19-induced myocarditis. It has been discovered that spike mRNA read of BNT162b2 vaccine is taken up by monocytes and macrophages, leading to the expression of spike proteins and subsequent inflammatory responses. Moreover, it has been found that spike proteins and their interactions with immunological receptors (59) cause the monocytes and macrophages to undergo pro-inflammatory shift (60), which is likely to be involved in pathogenesis of BNT162b2-MyoC. In our study, however, it was difficult to quantify spike mRNA reads or protein expression in individual cells (16 days after 1st vaccination) since they drastically decreased in terms of amount and hard to detect 7 days after vaccination (61). However, the other study revealed that it may persist for 60 days or longer (62). Therefore, further study is required on the effect of spike proteins on immune cells in BNT162b2-MyoC.

Overall, we analyzed classical monocytes in similar disease entities to explore the pathogenesis of vaccination-induced myocarditis. Although the analyzed data are from patients with COVID-19, myocarditis, or both, their molecular signatures differed in terms of AP-1 activity, and fatty acid metabolism, implying that the response of monocytes differs according to the stimulus. Moreover, molecular changes upon vaccination are different from the simple activation of monocytes. Here, we shed light on the most serious threat to public health worldwide by introducing classical monocytes as the key to understanding BNT162b2-MyoC.

Yet this study has a few limitations. Due to the scarcity of the case, the number of samples was limited. Further evaluation of PBMCs of other patients regarding the role of monocytes is necessary to confirm the characteristics of peripheral immune landscape specific for myocarditis after vaccination. Also, the patients with COVID-19-induced myocarditis and Kawasaki were treated with IVIG, which may have affected the signals observed in this study. Furthermore, in order to predict the presence or absence of vaccine side effects in individuals, an in-depth comparative analysis of datasets of vaccinees with and without side effects should be conducted. Lastly, datasets we used for our comparative research were mainly focused on COVID-19 inflammation and its impact on heart tissues, although pathogenesis of BNT162b2-MyoC may be involved with complications in lung or circulatory system as well. Future investigations into side effects of BNT162b2 of other vaccines for COVID-19 in general should also feature other major organ systems such as respiratory and endocrine system, where COVID-19 symptoms or side effects of BNT162b2 were reported. However, this study may take a meaningful first step towards understanding one of the serious side effects of BNT162b2.

## Data availability statement

The datasets presented in this study can be found in online repositories. The names of the repository/repositories and accession number(s) can be found below: Sequence Read Archive (SRA) (SRR19592869).

## Ethics statement

The study involving human participant was reviewed and approved by the Institutional Review Board (IRB) of Uijeongbu St. Mary’s Hospital (UC19TIDE0142). The patient/participant provided his/her written informed consent to participate in this study. Written informed consent was obtained from the individual for the publication of any potentially identifiable images or data included in this article.

## Author contributions

NH, YH, BY and SF conducted most of the research and drafted the manuscript. SB designed the research. KS and SK gathered clinical information of the patient. J-WK was involved in conceptualization of the study. BY, H-SA and SF were primarily involved in data collection, the development of the hypothesis, and developing the manuscript. All authors contributed to the article and approved the submitted version.

## Funding

This work was supported by the National Research Foundation (NRF-2021R1A2C2009749 and NRF-2018R1A5A2025079) from Ministry of Science and ICT, and the Korea Health Technology R&D Project (HR18C0012).

## Acknowledgments

We thank the financial supports by the National Research Foundation (NRF-2021R1A2C2009749 and NRF-2018R1A5A2025079) and Korea Health Technology R&D Project (HR18C0012), and the Seok-San Biomedical Science Scholarship, Yonsei University College of Medicine.

## Conflict of interest

The authors declare that the research was conducted in the absence of any commercial or financial relationships that could be construed as a potential conflict of interest.

## Publisher’s note

All claims expressed in this article are solely those of the authors and do not necessarily represent those of their affiliated organizations, or those of the publisher, the editors and the reviewers. Any product that may be evaluated in this article, or claim that may be made by its manufacturer, is not guaranteed or endorsed by the publisher.
