# Supplementary material for: Single-cell sequencing of PBMC characterizes the altered transcriptomic landscape of classical monocytes in BNT162b2-induced myocarditis
Source: Front Immunol. 2022 Sep 26;13:979188. doi: 10.3389/fimmu.2022.979188 (PMC9549039; doi:10.3389/fimmu.2022.979188)
Supplement: Supplementary file 1 [file DataSheet_1.pdf]

Supplementary Material

1 Supplementary Figures

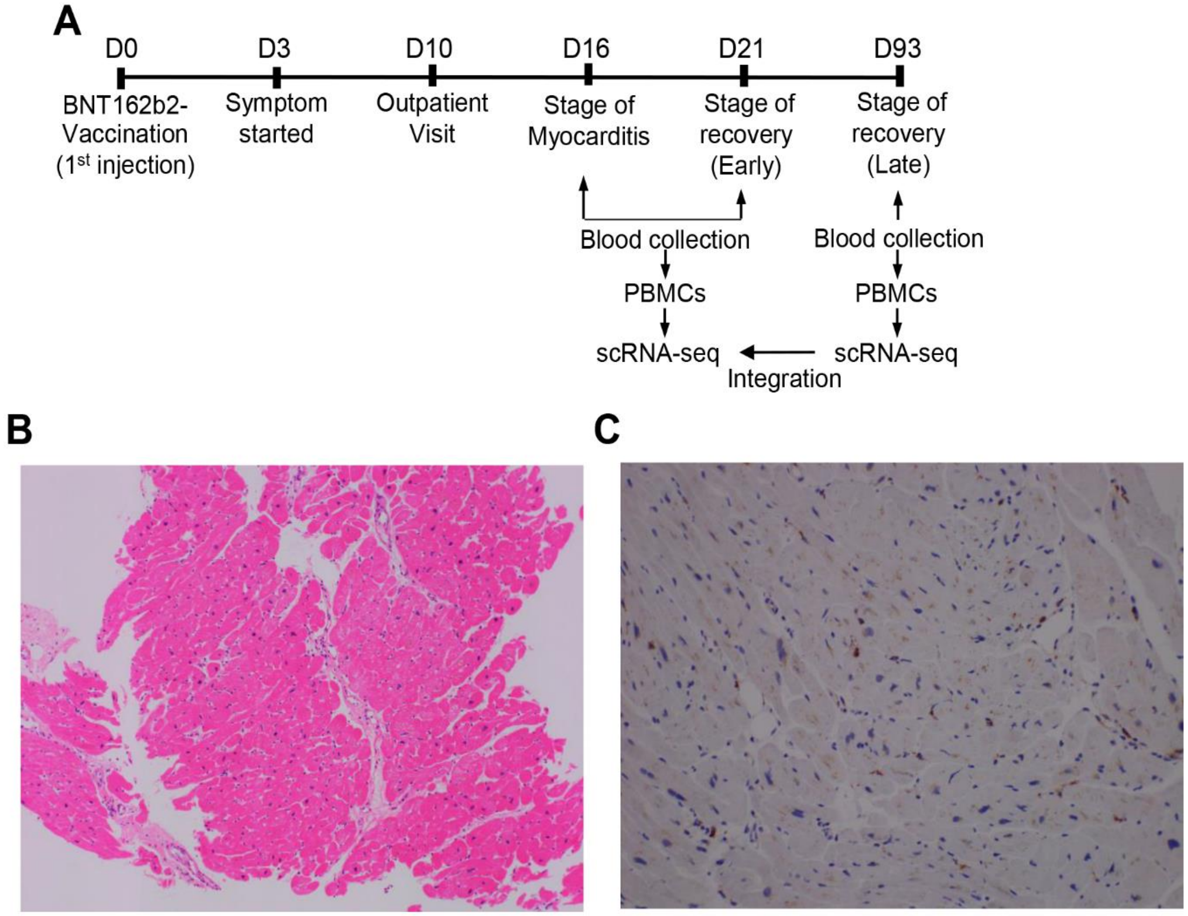

Supplementary Figure 1. Clinical symptoms of the patient with myocarditis induced by BNT162b2 vaccination **(A)** Time point of onset of myocarditis and blood collection **(B)** Endomyocardial biopsy showed active multifocal lymph histiocytic myocarditis with a few microfoci of myofiber eosinophilic changes and disrupted myocytes (H&E stain, ×200). The inflammatory infiltrates show mild extent and focal distribution of the lymphocytic type without fibrosis (left and right). **(C)** CD68 immunostaining showing widespread inflammatory infiltration of macrophages (in brown, ×200)

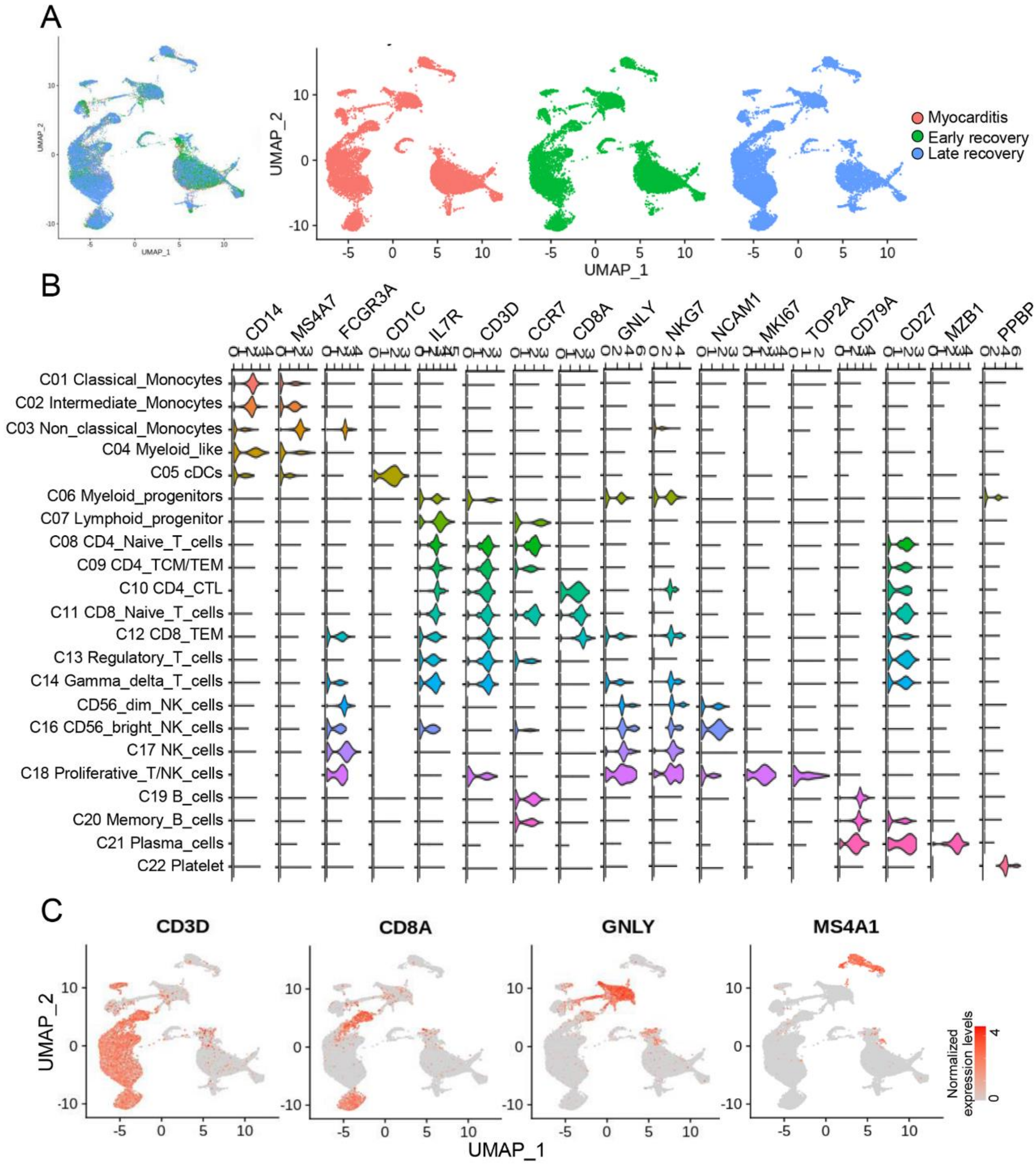

Supplementary Figure2. Annotation for Each Cell Type in the Integrated Dataset.

(A) UMAP showing each group of cells. **(B)** Violin plot showing the normalized expression of marker genes for immune cells in integrated dataset. **(C)** Normalized expression levels of marker genes for each immune cell type on a UMAP plot. CD3D (T cells), CD8A (CD8+ T cells), GNLY (Natural killer cells (NK cells)), and MS4A1 (B cells)

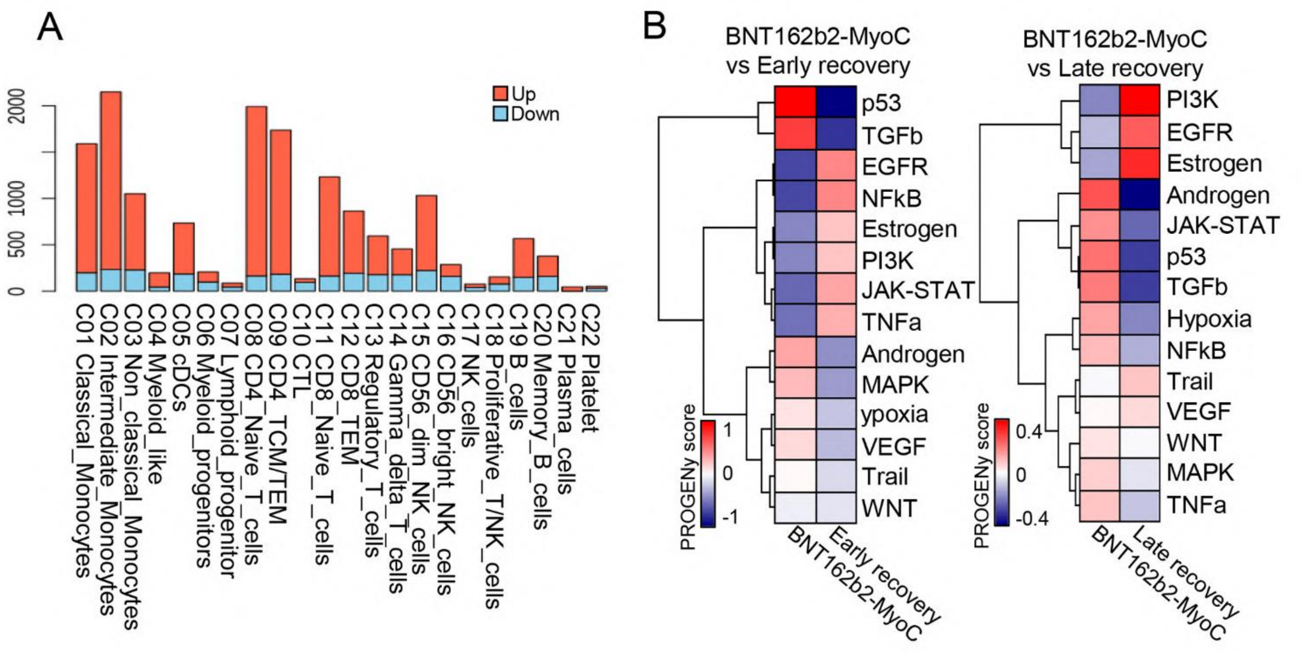

Supplementary Figure 3. Analysis of DEGs in Each Immune Cell Type in BTN162B2-MyoC Stage compared to Recovery Stage

(A) Numbers of differentially expressed genes (DEGs) within each immune cell type in the patient in BTN162B2-MyoC stage compared to late recovery stage (p-value < 0.01, $\log_2$ FC $\geq$ 0.15 or ≤ -0.15). Down; down-regulated; Up; up-regulated. **(B)** Heatmaps showing pathway-activities for classical monocytes in PBMCs from BNT162b2-MyoC stage versus early and late recovery stages, respectively.

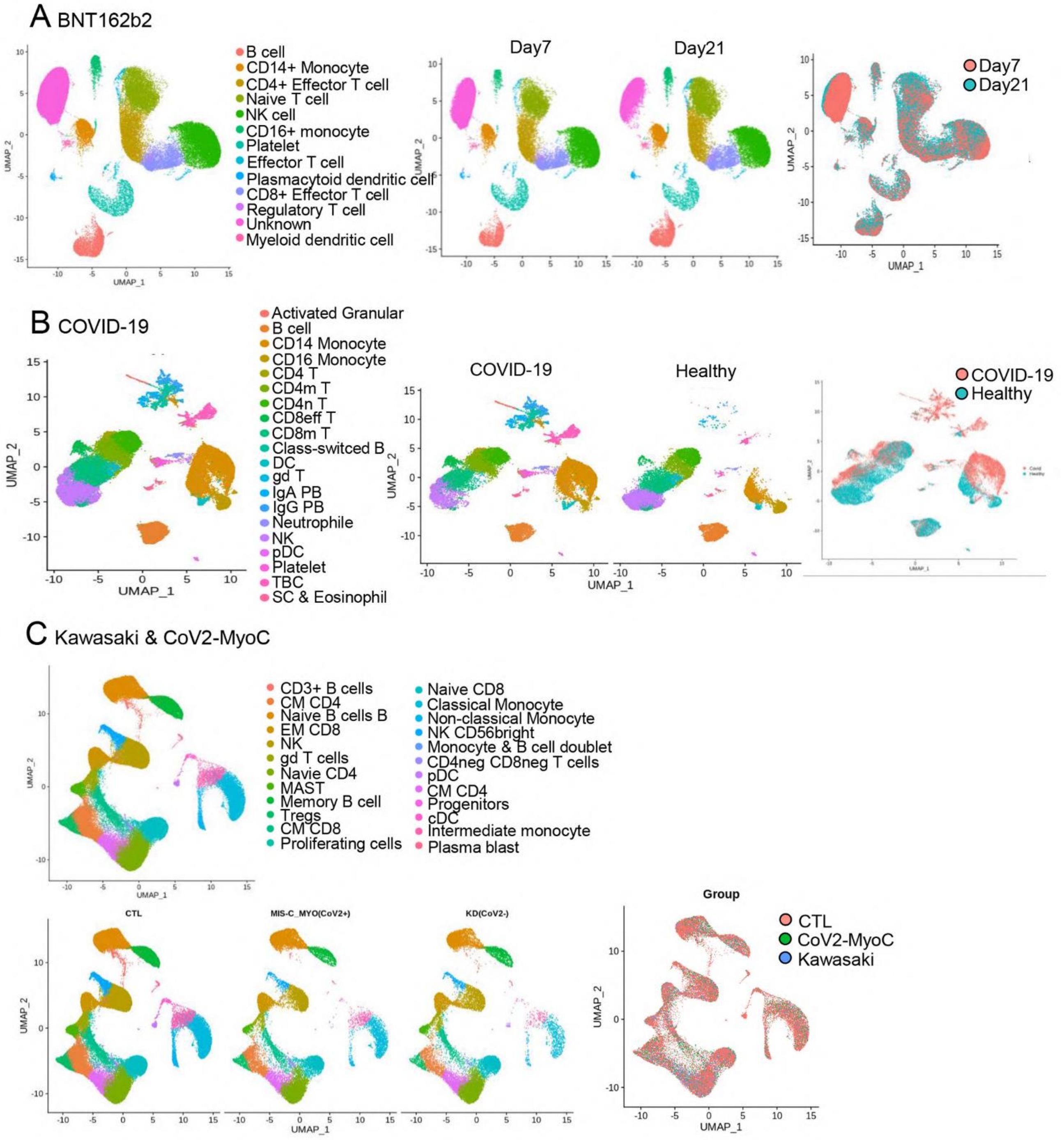

Supplementary Figure 4. Dimensional Reduction of Public Datasets

(A) UMAPs for BNT162b2 dataset colored according to cell type (left), divided by group (middle) and colored by group (right). Total number of cells per group: Day7 (n=39,597cells) and Day21 (n=32,398cells). **(C)** UMAPs for COVID-19 dataset colored according to cell type (left), split by group (middle) and colored by group (right). Total number of cells per group: COVID-19 (n=28,094cells) and Healthy (n=16,627cells). **(D)** UMAPs for dataset of patients with Kawasaki disease and patients with CoV2-MyoC colored according to cell type (up), divided by group (down, left) and colored by group (down, right). Total number of cells per group: CTL (Control, n=67,086cells) and CoV2-MyoC (n=21,799cells) and Kawasaki(n=17,826cells).

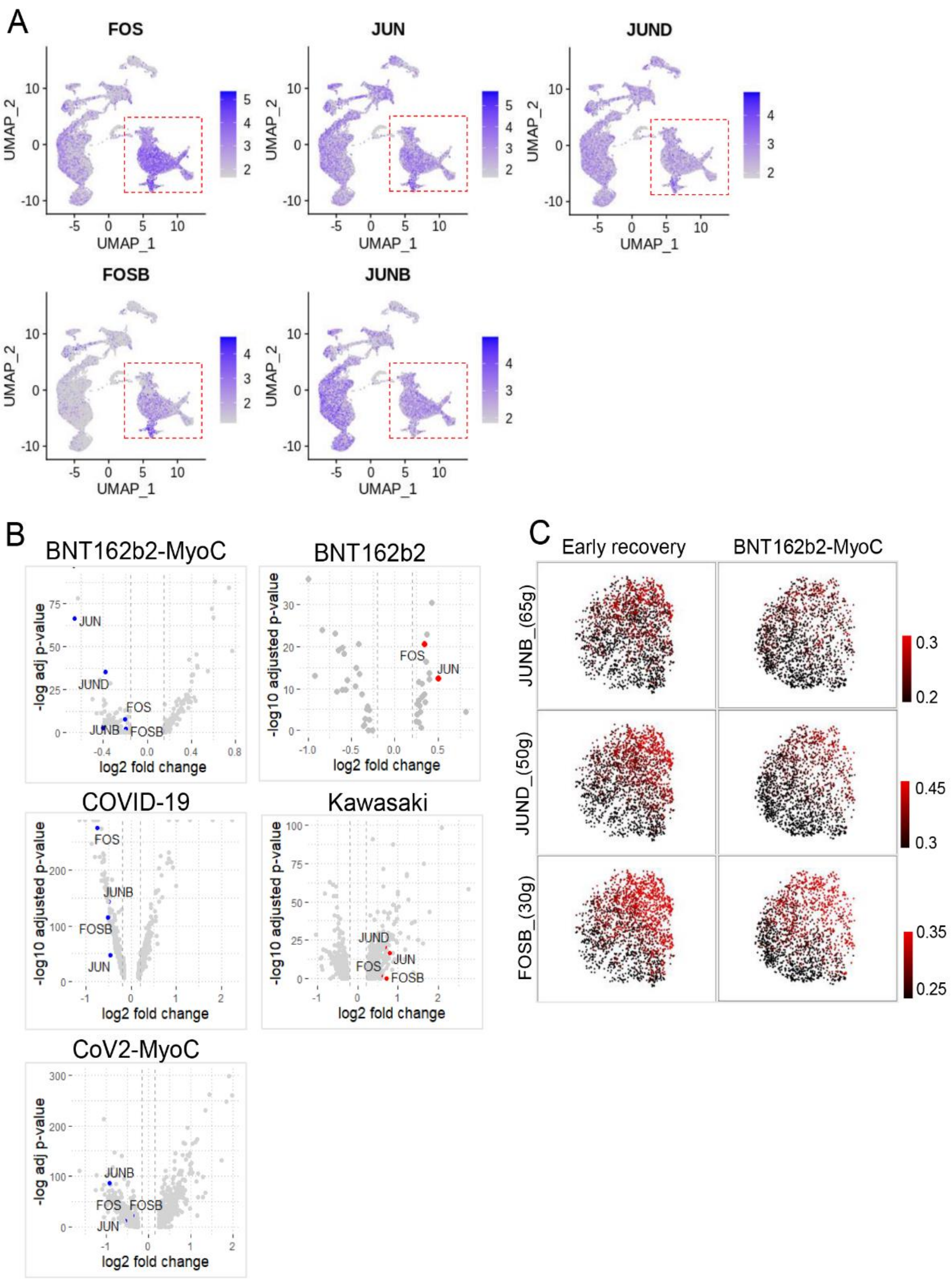

Supplementary Figure 5. Classical Monocytes featured Reduced AP-1 Activity during BNT162b2-MyoC phase.

(A) Normalized expression levels of AP-1 for whole cells on UMAP. Red box indicates the location of monocytes. **(B)** Volcano plot showing average expression levels of AP-1 in DEGs of classical monocytes in each dataset. Genes in upregulated DEGs (red) and in downregulated DEGs (blue) are labeled. **(C)** UMAP showing expression levels of JUNB, JUND and FOSB in classical monocytes in BNT162 b2-MyoC and in early recovery stage.

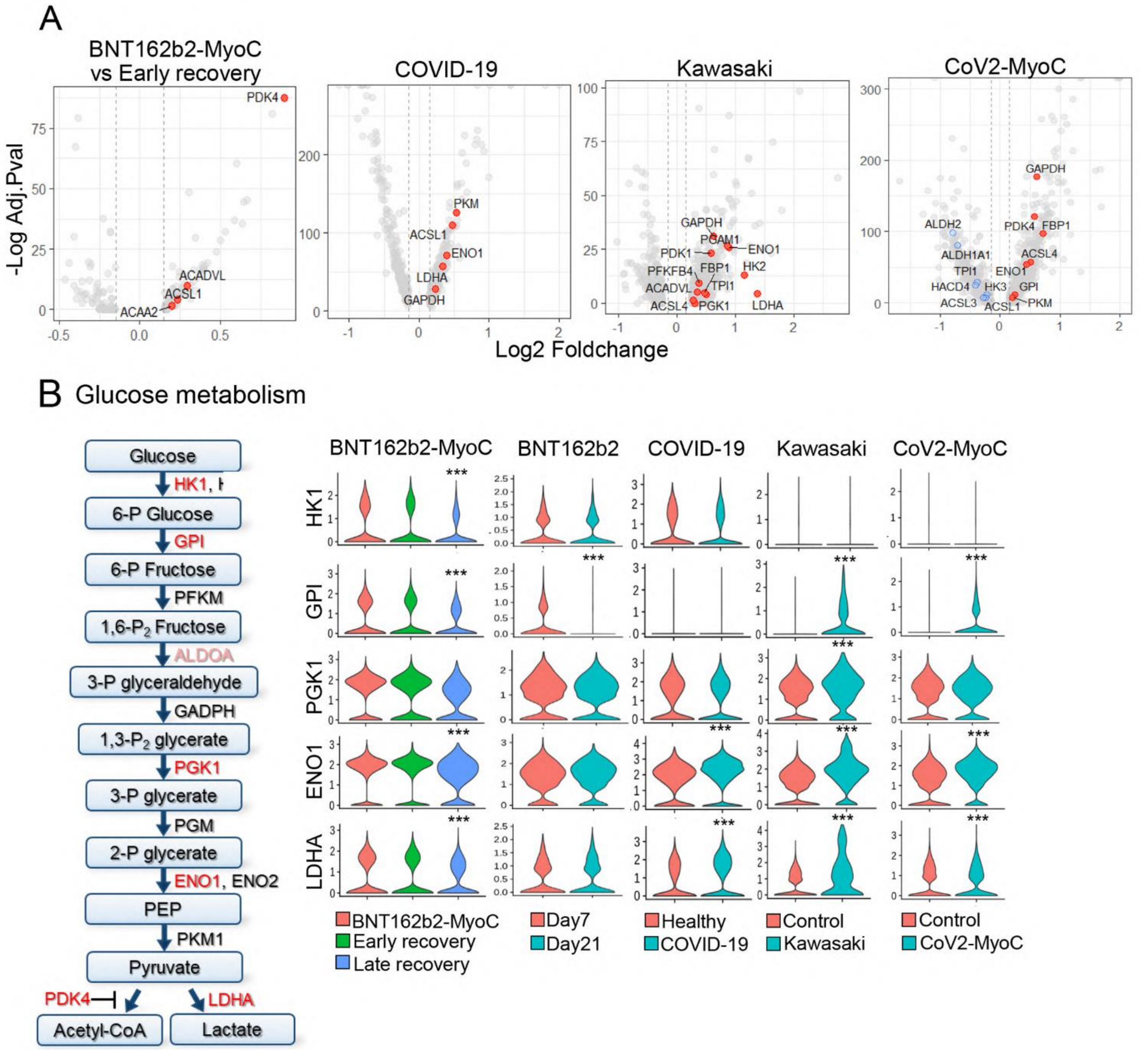

Supplementary Figure 6. Genes associated with Cellular Metabolic Pathways in DEGs of Classical Monocytes in Each Dataset.

(A) Volcano plots showing metabolism-related genes in DEGs of classical monocytes in each dataset (P-value < 0.01, log2 FC ≥ 0.25 or ≤ −0.25. red; up-regulated, blue; down-regulated genes). **(B)** A diagram of glycolysis pathway and violin plots showing expression levels of the glycolysis-related genes in classical monocytes.

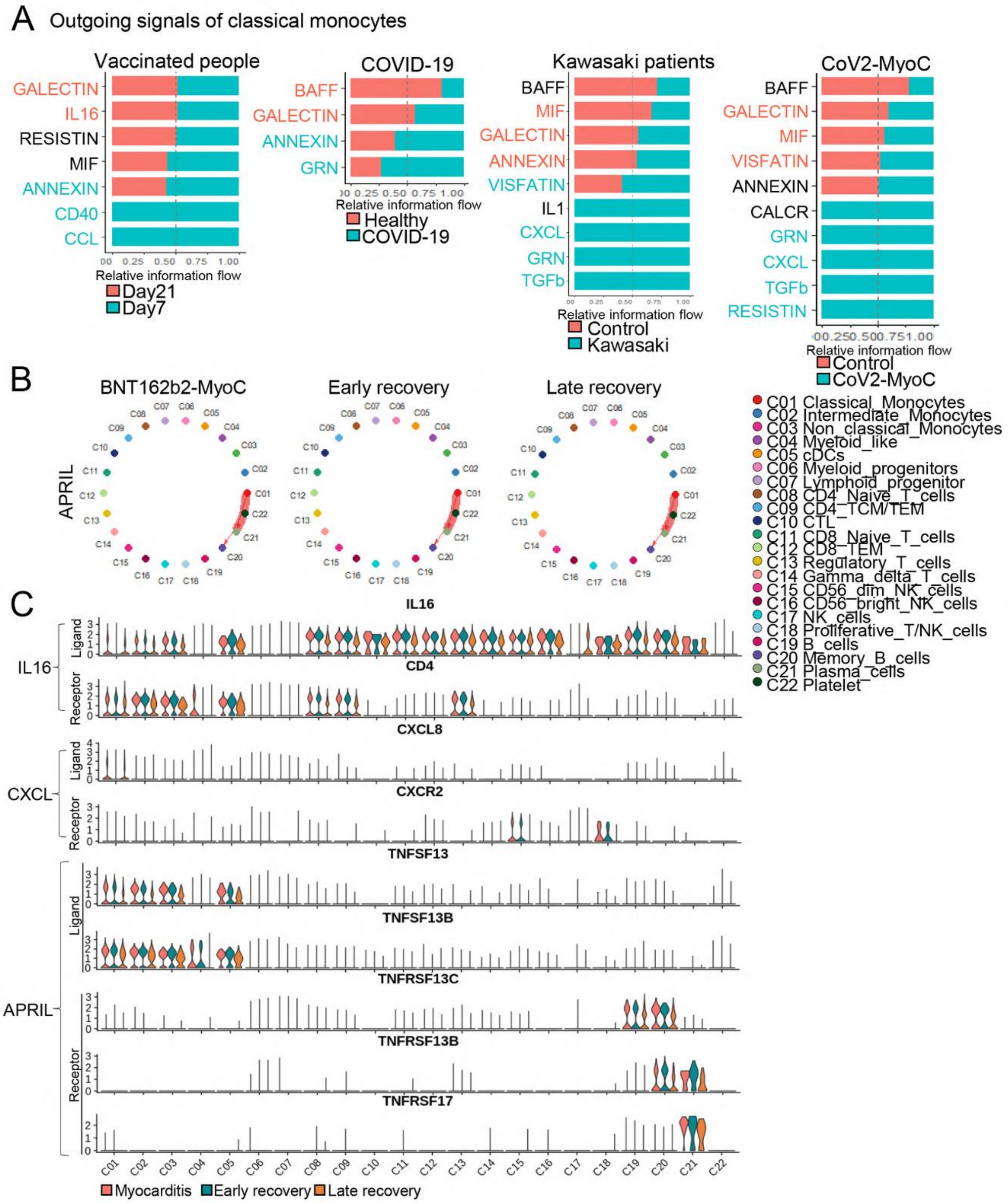

Supplementary Figure 7. Outgoing Signals of Classical Monocytes during BNT162b2-MyoC.

(A) Bar graphs showing the ranking of major outgoing signals of classical monocytes in each dataset. The rank of signals was based on differences in overall information flow, which is calculated by the total weights in the cellular network, in each group. **(B)** Circle plots showing the cell-to-cell communication network for APRIL signal entering to classical monocytes in BNT162b2-MyoC dataset. Arrows and edge color indicate direction (source: target). Edge thickness indicates the sum of weight key signals between populations. **(C)** Violin plots showing normalized expression levels of the ligand and receptor genes of cell-to-cell interactions in overall cell populations in BNT162b2-MyoC dataset.

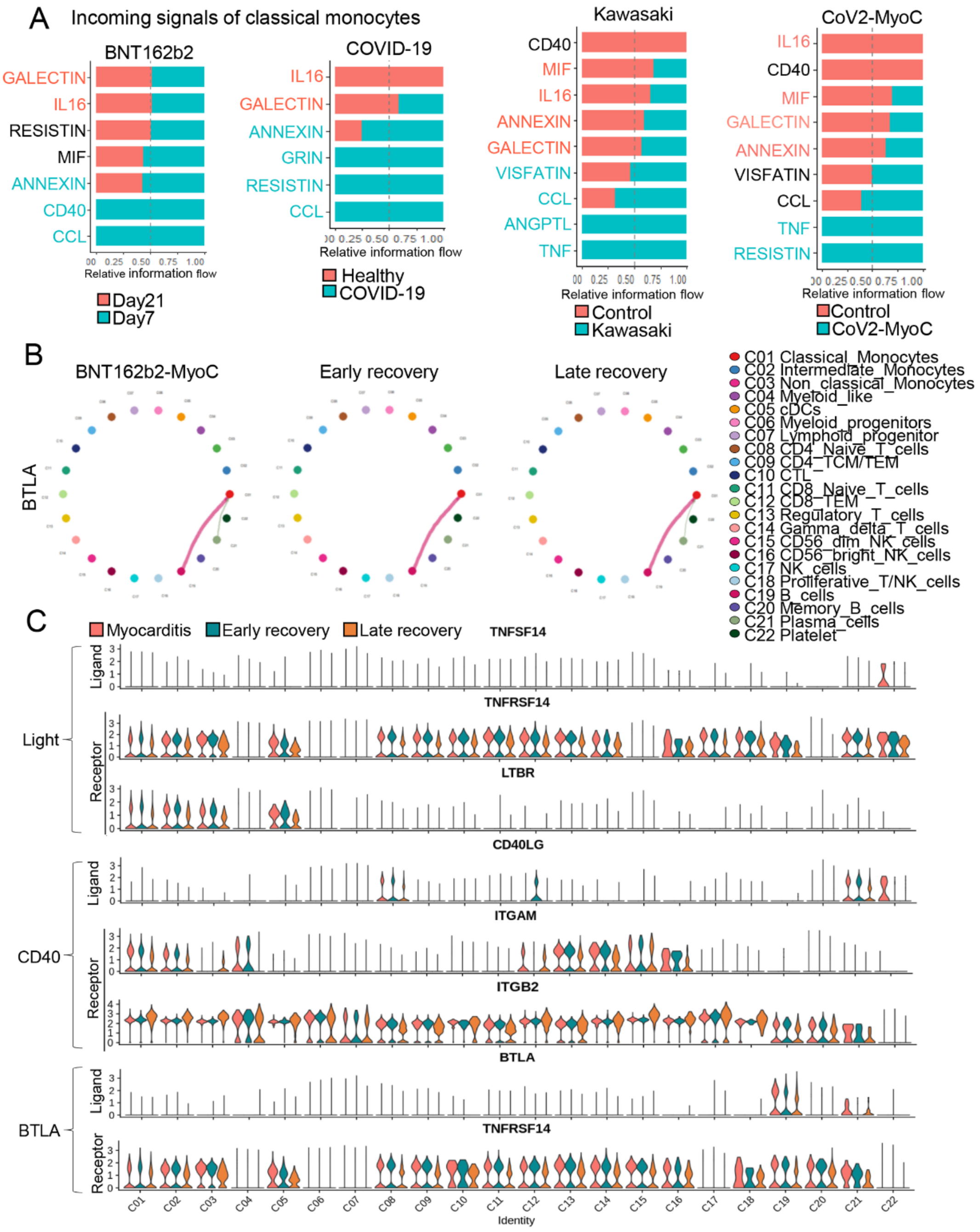

Supplementary Figure 8. Incoming Signals of Classical Monocytes during BNT162b2-MyoC

(A) Bar graphs showing the ranking of major incoming signals of classical monocytes in each dataset. The rank of signals was based on differences in overall information flow, which is calculated by the total weights in the cellular network, in each group. **(B)** Circle plots showing the cell-to-cell communication network for BTLA signal entering to classical monocytes in BNT162b2-MyoC dataset. Arrows and edge color indicate direction (source: target). Edge thickness indicates the sum of weight key signals between populations. **(C)** Violin plots showing normalized expression levels of the ligand and receptor genes of cell-to-cell interactions in overall cell populations in BNT162b2-MyoC dataset.
